# Owl-Neck-Spine-Inspired, Additively Manufactured, Joint Assemblies with Shape Memory Alloy Wire Actuators

**DOI:** 10.3390/biomimetics8010117

**Published:** 2023-03-11

**Authors:** Robin Löffler, Stephan Tremmel, Rüdiger Hornfeck

**Affiliations:** 1 Department of Mechanical Engineering and Building Services Engineering, Nuremberg Institute of Technology, Kesslerplatz 12, 90489 Nuremberg, Germany; 2 Engineering Design and CAD, University of Bayreuth, Universitätsstraße 30, 95447 Bayreuth, Germany

**Keywords:** biomimetic innovation, additive manufacturing, shape memory alloys, resource efficiency, sustainability

## Abstract

Nature provides a considerable number of good examples for simple and very efficient joint assemblies. One example is the enormously flexible cervical spine of American barn owls, which consists of 14 cervical vertebrae. Each pair of vertebrae produces a comparatively small individual movement in order to provide a large overall movement of the entire cervical spine. The biomimetic replication of such joints is difficult due to the delicate and geometric unrestricted joint shapes as well as the muscles that have to be mimicked. Using X-ray as well as micro-computed tomography images and with the utilisation of additive manufacturing, it was possible to produce the owl neck vertebrae in scaled-up form, to analyse them and then to transfer them into technically usable joint assemblies. The muscle substitution of these joints was realised by smart materials actuators in the form of shape memory alloy wire actuators. This actuator technology is outstanding for its muscle-like movement and for its high-energy density. The disadvantage of this wire actuator technology is the low rate of contraction, which means that a large length of wire has to be installed to generate adequate movement. For this reason, the actuator wires were integrated into additively manufactured carrier components to mimic biological joints. This resulted in joint designs that compensate for the disadvantages of the small contraction of the actuators by intelligently installing large wire lengths on comparatively small installation spaces, while also providing a sufficient force output. With the help of a test rig, the developed technical joint variants are examined and evaluated. This demonstrated the technical applicability of this biomimetic joints.

## 1. Introduction

**Figure 1:**
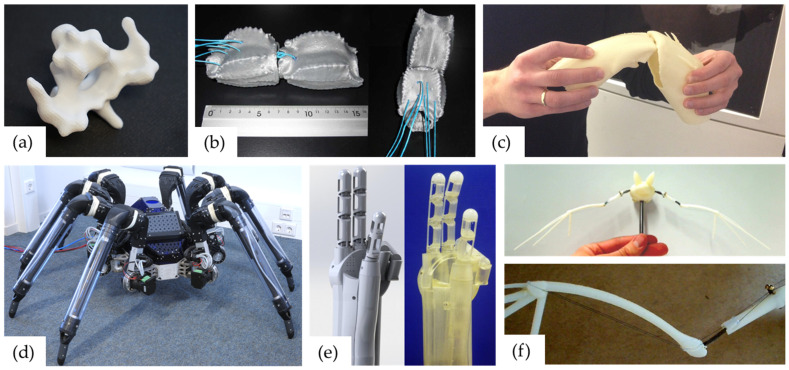
Three-dimensionally printed analytical models of biological models are shown in the top row and technically converted, also 3D-printed, joints are shown in the bottom row: (**a**) owl cervical vertebra; (**b**) scorpion tail joint [7]; (**c**) spider leg joint [6]; (**d**) “bionic” spider [6]; (**e**) ”bio-inspired” hand [8]; (**f**) ”bio-inspired” bat (BATMAV) with SMA actuator wires [9].

Additive manufacturing (AM) presents itself as an excellent manufacturing technology for biologically inspired mechanical assemblies [1,2,3,4]. All AM technologies have the layered part building process in common. For example, either heat-liquefied plastic is layered on top of each other through a nozzle (Fused Deposition Modelling—FDM), parts are produced by sequentially layering plastic or metal powder in a powder bed and then melting it by laser (Selective Laser Sintering—SLS and Selective Laser Melting—SLM), or resins are selectively cured layer by layer with ultraviolet light (Stereolithography—SLA). In particular, AM stands out from conventional manufacturing techniques due to the great geometric design freedom, the wide variety of materials, the high production speed (rapid prototyping), and the constant production costs from a quantity of one [5]. It is thus possible, for example, to print biological joints, joint assemblies, or bone structures in great detail for analysis purposes [6,7] (Figure 1a–c) or to produce biomimetic designs for technical applications with high mechanical loads [6,8,9] (Figure 1d–f). Even the flexible membrane of the spider’s leg in Figure 1d is printed using SLS technology. For highly stressed zones within the components, either thinner wall cross-sections in the form of a topology optimisation or local density differences through different infill patterns can be applied to save material. Through these possibilities, biomimetic product development can be optimised in terms of quality, speed, and, in particular, practical feasibility.

In combination with smart materials, in the case presented in this paper of wires made of shape memory alloys (SMA), the advantages mentioned come into their own. Actuators made of SMA generate their movement through a reversible phase transformation from martensite to austenite [10,11]. This phase transformation causes the actuator to remember a trained geometric shape, which provides it its name [12,13]. For example, it is possible to shorten a 100 mm long and 0.381 mm in diameter nickel–titanium (NiTi) wire by heating it to 78 °C to a length of about 95 mm and thereby lift a weight of 2000 g. The weight of the wire actuator is only 0.074 g (0.395 g with ring cable terminals) [14]. It is possible to make this muscle-like movement of the SMA wire actuators optimally usable for joint assemblies by means of AM carrier components. The problem of the difficult combination of high-force, large-stroke, and small installation space can thus be addressed. The comparatively low stroke of the SMA wire actuators is often cited as the main difficulty of this actuation technology [15]. This problem can be tackled by appropriately fitting the SMA wire actuators in a three-dimensional space. The highest energy density among the known actuator principles, which results in an enormous force with low self-weight, is the crucial factor [16,17]. A suitable mechanical transmission, which leads to a larger stroke with less force, can therefore be used.

The need to design such joint assemblies arose during research into a biomimetic joint robot arm (hyper-redundant robot) based on the model of the cervical spine of American barn owls [18]. The delicate vertebral structures with the internal supply channels of the owl’s neck [19] had to be technically abstracted and reproduced. In particular, the actuator selection and integration posed the greatest challenge. Only by using the aforementioned SMA wire actuators was it possible to implement the motions of the biological model of the barn owl within the framework of the tight installation spaces. The limitation of the use of the SMA wire actuators due to their small contraction was of less importance, as in the case of the owl’s cervical spine, many small movements of the individual cervical vertebrae produce a large overall movement. At the same time, the use of little raw material in AM and SMA wire actuators made it possible to achieve a resource-efficient arrangement of biomimetic joints.

The framework for the development of the biologically inspired articulated robot arm is the guideline 6220 (Biomimetics: Fundamentals, conception, and strategy) of VDI (Verein Deutscher Ingenieure—Association of German Engineers) [20]. This guideline defines the requirements for a biomimetic system and presents basic information on biomimetic development processes. The requirements are as follows: A Biological Model is available, The Working Principle is abstracted and transferred into a Technical Application and A Prototype of the Implementation is available. These requirements are used to enable the development of the articulated robotic arm, shown to be comparable with other biomimetic projects.

The research presented in this paper includes the combination of the abstracted owl neck vertebrae for biomimetic application, AM, and smart materials actuators in the form of SMA wire actuators. More specifically, joint assemblies with different mechanical transmissions are presented. These joint assemblies were manufactured and examined by means of experiments. In the process, the prevailing disadvantage of SMA wire actuators in the form of the low contraction had to be compensated by the enormous potential of the highest energy density of SMA wire actuators [16,17] in combination with AM, so that generally applicable joint assemblies could be developed. The original idea for these joints was the cervical spine of the American barn owl with its 14 individual joints [19]. Based on the different joint assemblies, the potential of combining these topics is presented and made usable for other engineers and researchers in such a way that joint assemblies for the most diverse applications can be designed within a shortened development time. In particular, the focus is on the efficient use of resources through the material-saving potential of AM technology and SMA wire actuators [21]. This eliminates the use of conventional, heavy drives in the form of electric motors, hydraulics, or pneumatics.

**Figure 2:**
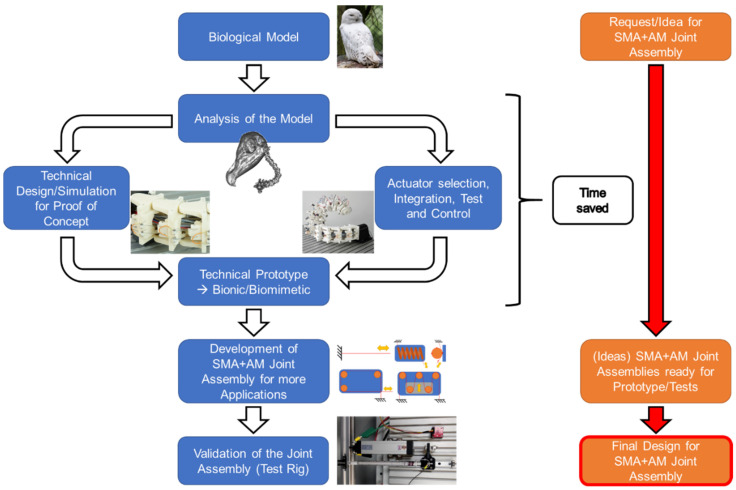
Flowchart illustrating the research process (left/blue) compared to the streamlined development process using the research results (right/orange).

Figure 2 shows the development of the biologically inspired joints as a flowchart. The chronological representation clearly illustrates the entire development process, starting with the biological inspiration, through the biomechanical investigations and the motion simulation, to the first technical prototype and the resulting joints. In particular, the greatly reduced development process through the research presented in this article is illustrated on the right side of the figure. At the same time, the flowchart illustrates the structure of this paper.

## 2. Materials and Methods

### 2.1. Transfer of the Biological Model to Technical Joint Assemblies

**Figure 3:**
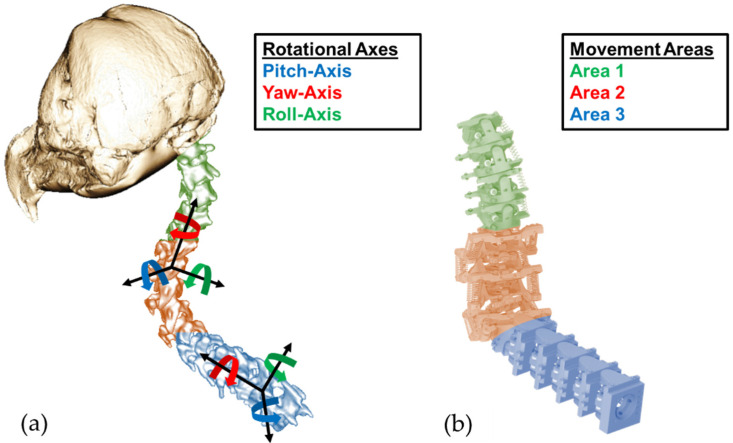
(**a**) Micro-CT image of the owl’s neck spine with colour-coded rotational axes and movement areas. (**b**) Biomimetic model with analogue-marked movement areas.

The necessary biological motion data as a foundation for the development of the joint assemblies could be obtained, evaluated, and abstracted through the cooperation with the RWTH Aachen, Institute of Biology II (Zoology). There, the data were collected by analysing dissected American barn owls as well as through X-ray and micro-CT images of the animals. The data were then further analysed in the form of 3D models. In this way, the great mobility of the cervical spine of American barn owls was proven and quantified. In particular, Krings et al. [22] described the subdivision of the cervical spine into three relevant areas of motion (see the coloured areas in Figure 3). Depending on the range of motion, the individual movements of the cervical vertebrae take place around different axes of rotation. It was also found that saddle joints occur for the most part. Only the last two joints in the direction of the head differ from this. The second-last joint is a pivot joint. The last joint in front of the owl’s head is a ball and socket joint [19,22].

These datasets served as the basis for developing the technically abstracted joint assemblies from the biological model of the individual joints. For this purpose, slightly different articulation angles within the motion areas were adapted to each other so that only identical pairs of vertebrae were installed in each of the three motion areas. For example, this is clearly visible in the blue-marked area 3 in Figure 3. Compared to the biological vertebrae on the left side, the technical vertebrae on the right side are exactly the same, which greatly simplified the production and following control concept. In addition, the saddle joints, which enable the movement of the respective pairs of vertebrae around two axes of rotation, were reshaped into two successively linked rotary joints. This allowed a technical design in the form of a fixed bearing of the axes of rotation, whereby a doubling of the number of vertebrae occurred. After the technical design of the cervical spine, its overall mobility was simulated and successfully verified with the help of inverse kinematics [23] in the development environment of Unity3D 2018.4.11f1 (Unity Technologies, San Francisco, CA, USA). The procedure for the simulative verification of the biological movement is described in [18] using a specifically developed method.

### 2.2. Manufacturing of the Biomimetic and Technical Joints

Different manufacturing processes were used to produce the biomimetic vertebrae of the robot arm and the technical joints of the test rig. The choice of process depended on the geometry and the mechanical as well as the thermal requirements of the parts. For example, milled components made of aluminium were selected for attaching measuring instruments to the test rig, as the force and stroke measurements are affected by deformed parts. The basic structure of the test rig was also made of aluminium profiles. Due to the robust setup, only the SMA wire and joint component deformations should influence the measurement. The actual biomimetic joint assembly parts were made of ABS-M30i using the FDM printing process on a Fortus 400mc Large (Stratasys Ltd., Rehovot, Israel). This was particularly beneficial for the prototype due to the free geometric design possibilities and the rapid manufacturing speed. The mechanical strength and temperature resistance of the components produced in this way are sufficient, taking into account the correct part orientation in the building chamber, because of the anisotropic mechanical properties. Referring to the manufacturer’s website and data sheet, the manufacturing parameters of the components and the relevant material characteristics are described in Table 1 [24,25]. The mechanical and thermal characteristics of the support material are not described because it was removed after the printing process.

The deflection components in contact with the SMA wire actuators were made of polytetrafluoroethylene (PTFE). PTFE has a comparatively high heat stability and very low friction with the SMA wires. Since the expected temperatures of the SMA wires are 70–100 °C, PTFE is generally sufficient and recommended, as PTFE also insulates electrically. During the preliminary and prototype tests, which consisted of up to 100 cycles, no sinking of the wires into the PTFE deflection components was observed. The PTFE components were manufactured conventionally, via turning or milling.

### 2.3. Shape Memory Effect and Characteristics of the SMA Wires Used

**Figure 4:**
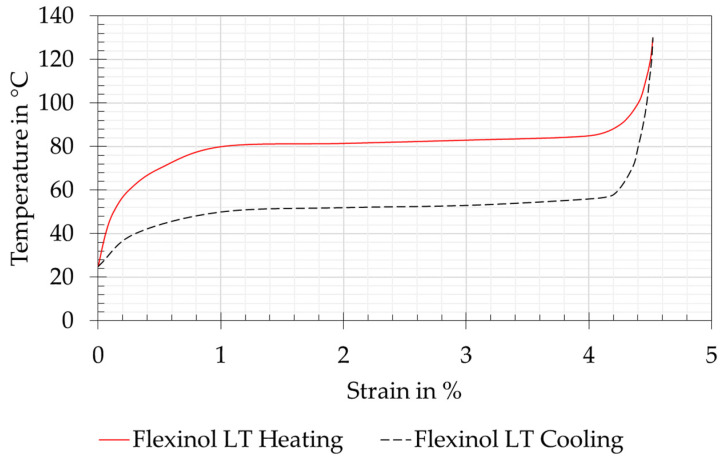
Relationship of the temperature to the strain of Dynalloy Flexinol Low Temperature (LT) wire during heating and cooling. Reproduced from [14].

SMA wire actuators were used to actuate the biomimetic joints in the prototype of the technical owl’s neck and for the measurements in the test rig. More precisely, these were Flexinol Low Temperature (LT) wires (Dynalloy Inc., Irvine, CA, USA), which are made of a NiTi alloy. The transition temperatures are described in [26] and listed in Table 2. The specifications for the contraction and cooling times are provided by the manufacturer. The contraction time referred to a complete transformation of the wire with the given information on the electrical power. The cooling time served as an approximate reference value at a wire temperature of 70 °C. Figure 4 shows the corresponding strain and temperature curve provided by the manufacturer [14].

The Flexinol SMA wire actuators have the advantage that they are supplied ready for use and therefore additional training, as is the case with other commercially available wires, is not necessary [14]. They can be used directly with the extrinsic two-way shape memory effect, which means that the SMA wire can remember its shape at both high and low temperatures. A mechanical force is still needed to reset the wire. Depending on the required pulling force, either 0.254 mm (0.01 in) or 0.381 mm (0.015 in) diameter wires were used in the biomimetic joint assemblies of the prototype. Wires with a diameter of 0.254 mm (0.01 in) were used in the test rig. The exact data of the SMA actuator wires are provided by the manufacturer and can be found in [14].

According to the manufacturer, several tens of millions of cycles are possible if the values provided in [14] are maintained. If the given operating temperature is exceeded, the number of cycles may be greatly reduced at first and the wire actuator may be damaged in the long run. Since neither the articulated robot arm nor the test setup of the technical joint assemblies is intended to achieve such a high number of cycles, the specified maximum forces are exceeded in some cases. The targeted number of cycles is in the range of a few thousand contractions.

The SMA wire actuators were formed into loops at both ends and crimped with the help of ring cable terminals. This formed a force-fit connection between the SMA wire and the ring cable terminal. Fastening to the part was achieved by means of screw connections, which created a further frictional connection between the loop-shaped wire, the screw head, and the contact surface of the ring cable terminal. If the wire had slipped, this could be checked visually at the ring cable terminal. In the previous tests, no slippage could be observed. The crimping procedure is based on Czechowicz [10], the manufacturer’s specifications [14], and VDI 2248 [21].

### 2.4. Biomimetic Joint Assemblies

**Figure 5:**
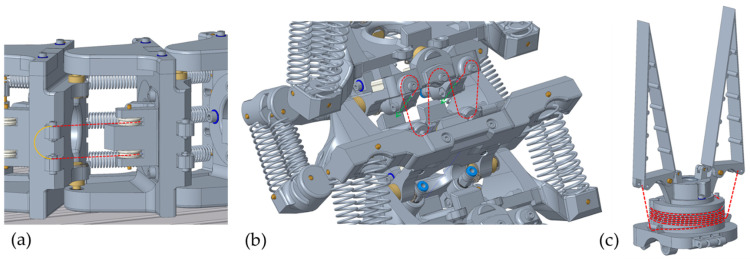
(**a**) Roll joint assemblies in the lower area. (**b**) Pitch joint assemblies in the middle area. (**c**) Fin Ray gripper on the end effector. The SMA wire actuators are shown as red dashed lines.

Through the transfer described in Section 2.1 from the biological model to technical joint assemblies, it was possible to develop a total of five biomimetic joint assemblies for the three cervical vertebrae areas as well as a gripper mechanism for the end effector. Of the total of six mechanisms, three joint assemblies were fundamentally different. These are shown as 3D models in Figure 5. The other three joint assemblies were analogously designed and are therefore not described separately. The information on the required wire lengths and forces resulted from geometric, weight, and centre of gravity analyses of the CAD model. More precisely, these data were used to calculate the distances between the points of force engagement and the axes of rotation. The pose that was extended the greatest distance in relation to the axis of rotation was always used as the reference point. The return forces of the antagonist springs were included as a further contributing factor. These forces were needed to pull the SMA wires in their cold martensite form back to the initial length. This reduced the actuation force of each vertebra. Appropriate safety factors were included for the weight deviations of the physical prototype. The results of these calculations for the required wire lengths and diameters of the corresponding vertebrae are described below. A detailed description of the calculations is not provided, as the articulated robot arm presented in this section serves as inspiration for the technical joint assemblies described in 3.2, which are the focus of the article. The results of the force, displacement, and power measurements of this technical joint assemblies are described in more detail below.

#### 2.4.1. Joint Assembly for the Roll Movement in the Lower Area (Area 3)

In Figure 5a, the joint assembly for generating the roll movement in the lower area of the owl’s neck spine can be seen. Five of these were arranged successively, each of which generated ±22.5° as a single movement. This created a roll movement of ±112.5° in the entire area. As in the biological model, this resulted in the curved shape of area 3 of the cervical spine and the lateral offset of the following areas compared to the vertical centre axis [22]. The theoretically necessary force of 46.4 N was generated by the parallel installation of two SMA wire actuators with a diameter of 0.381 mm. The movement was reset by three parallel tension springs. In order to be able to provide the necessary wire length of the SMA actuators of 1500 mm for these joint assemblies, the wires were installed underneath the worktable and guided directly to the respective vertebrae by means of a Bowden cable arrangement. The Bowden cable consisted of the inner SMA wire, which is enclosed by a PTFE tube and a long tension spring. The Bowden cable was mechanically connected by means of pneumatic connectors. As shown in Figure 5a, a pulley was attached to the vertebra to align the SMA wire, which guides the wire to the fastening screw of the following vertebra. For easier electrical wiring, the mechanically parallel FGL wires were electrically connected in series. This means that the positive and negative poles are located underneath the worktable.

#### 2.4.2. Joint Assembly for Pitch Movement in the Middle Area (Area 2)

In Figure 5b, the second significantly different variant for installing the replacement muscles in the form of SMA wire actuators for producing the pitch movement of the middle section can be seen. Five joint assemblies were arranged in sequence for a possible total movement of ±125°. This movement is mainly for tilting and raising the end effector. It should be noted that a large negative movement resulted in a collision with the work table during this setup of the robot arm. Each of the individual joint assemblies generated a movement of ±25° and had to be able to move a force of 129.4 N in theory. To generate the force, an SMA wire with a diameter of 0.381 mm was installed in each joint assembly in an arrangement comparable to a factor pulley. Due to the arrangement with four movable wires, analogous to ropes in a pulley block, the load on the respective wire segments was reduced by a factor of four, where, at the same time, four times the wire length had to be installed to generate the movement of 23 mm. With a necessary safety factor, an SMA wire with a total length of approximately 2650 mm had to be installed. Analogous to the variant shown in Figure 5a, the SMA wires installed in this section were guided under the work table by means of Bowden cables in order to install a greater wire length there. The difference here is that only one wire was installed per joint assembly. This was guided upwards to the vertebra once, installed there as shown, and then guided back under the worktable. The wire was reset via parallel tension springs on both sides of the respective pair of vertebrae.

#### 2.4.3. Joint Assembly for the Fin Ray Gripper

The third variant can be seen in Figure 5c. This variant was used for the gripping movement of the Fin Ray gripper at the end effector. The basic function of the biomimetic Fin Ray gripper [27] is not part of this research project. In order to achieve an energy-free holding position of the gripper, the gripping movement of the two jaws was realised with an internally installed tension spring. A 0.25 mm SMA wire actuator was used only for the quick opening movements of the gripper. This wire was wound spirally around the base body of the gripper in a 0.5 × 1 mm PTFE tube. This allowed a wire length of approximately 1000 mm to be fitted into the available installation space and a theoretical opening angle of 50° to be produced with sufficient holding force for a weight of 150 g. The use of a single SMA wire was sufficient for the assembly. This wire was attached with its two ends to the force engagement points of the gripper jaws. The electrical cables could be guided through the hollow inside of the gripper to the channel in the technical cervical spine. This resulted in a very compact mechanical and electrical design with a gripper weight of only 27 g.

### 2.5. Test Rig and Technical Joint Assemblies

The biomimetic joint assemblies described in Section 2.4 were further simplified, manufactured, installed in a specially developed test rig, and analysed in pre-tests. Friction and force losses in the deflection points as well as the general usability of the joint assemblies and the possibility of compact design were examined.

**Figure 6:**
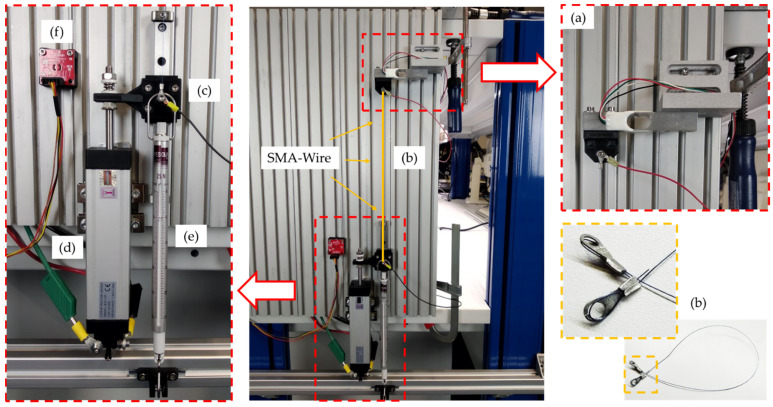
Vertical test rig for pre-tests: (**a**) load cell Sauter CK 6-0P1 (KERN & SOHN GmbH, Balingen-Frommern, DE); (**b**) 250 × 0.25 mm SMA wire actuator; (**c**) connecting platform; (**d**) linear potentiometer KTC 75 P (Ixthus Instrumentation, Towcester, UK); (**e**) tension spring balance Medio 25 N (PESOLA Präzisionswaagen AG, Schindellegi, CH) to generate the necessary counterforce; and (**f**) temperature sensor TMP117 (SparkFun Electronics, Boulder, CO, USA).

**Figure 7:**
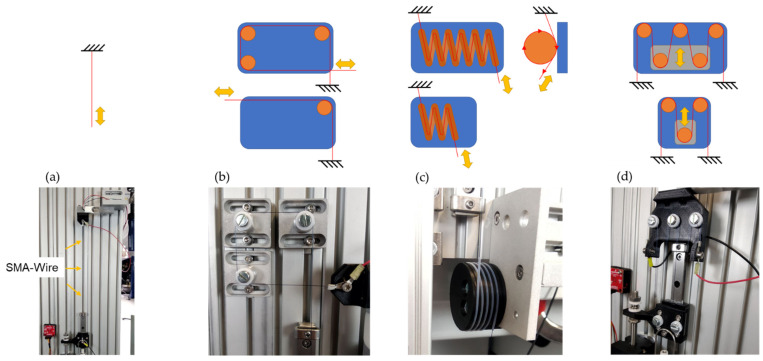
Different deflection variants in the sketch are shown in the top row and the final manufactured joint assemblies in the bottom row. (**a**) Straight clamped SMA wire for reference measurement; (**b**) deflection variant using PTFE pulleys with 3 × 90° and 1 × 90° deflection; (**c**) helix deflection in PTFE tube with long helix and short helix and (**d**) pulley block with PTFE pulleys with big pulley and small pulley.

In the centre of Figure 6, the vertically oriented mounting plate and the test rig assembly can be seen. Basically, the measurement setup always included a load cell at the upper end of the test rig (a), the SMA wire actuator clamped in between (b), a connecting platform between the SMA wire (c), the linear potentiometer (d), and a counterweight in the form of a tension spring balance at the lower end of the test rig (e). The arrangement of the SMA wire was variable due to different deflection variants. Thus, different wire lengths could be fitted and tested in different installation designs. Figure 7 shows a straight clamped SMA wire actuator as a reference measurement and three deflection variants, analogous to the vertebra assemblies in the biomimetic articulated robot arm described in Section 2.4. The three deflection variants also include two sub-variants each. For all measurements, the 0.25 mm diameter SMA wires, described in Section 2.3, were used in lengths of 250 mm, 450 mm, and 750 mm. The PTFE pulleys shown were used in combination with ball bearings to minimize rolling friction. A detailed description of the deflection variants is shown in Table 3. In order to obtain repeatable results, a new SMA wire was used for each of the measurement series of the variants. All measurements were carried out at room temperature in the range of 21–24 °C. 

**Figure 8:**
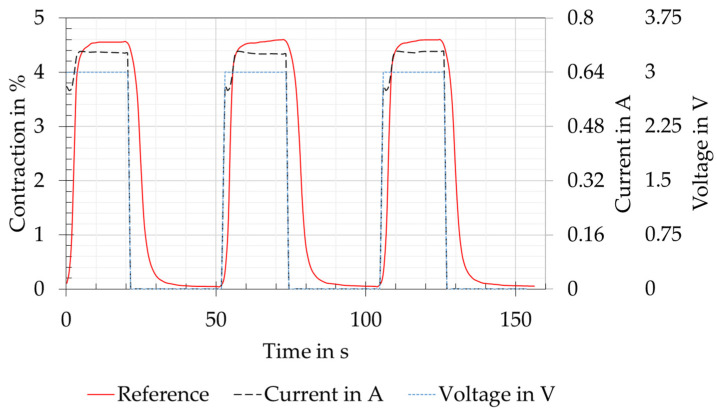
Relationship between the electric current, the voltage, and the contraction of the SMA wire actuator on the basis of a reference measurement with a clamped straight wire. A constant voltage of 3 V was applied for each contraction cycle of the SMA wire.

As can be seen in Figure 8, the SMA wire was supplied with power at a constant voltage for each contraction cycle. The power was provided by a E36313A laboratory power supply unit (Keysight Technologies, Santa Rosa, CA, USA). The current results from the relationship between power and resistance. In the process of the current increase, a slight drop could be seen at about 0.6 A. This results, as described by Lewis et al. [28], from a short increase in resistance with increasing temperature. This causes the power and the temperature to drop for a short time. After less than four seconds, the current stabilised again and increased to the maximum. This behaviour results from the decreasing resistance as the phase transformation and thus the contraction of the wire is continued. The geometric change, therefore, affects the resistance more than the temperature [29,30]. This behaviour could be seen in all test versions and could be tolerated for the pre-tests, as the maximum forces and contraction were reached within an acceptable time. For further tests, the aim should be power control, as this can ensure a constant temperature during the phase transformation [28].

Data collection was conducted with a parallel system consisting of an Arduino Uno (Arduino, New York, NY, USA), a 34460A bench multimeter (Keysight Technologies, Santa Rosa, CA, USA), and an E36313A laboratory power supply unit (Keysight Technologies, Santa Rosa, CA, USA). The data from the Arduino Uno were collected with a self-developed software based on MATLAB (The MathWorks, Inc., Natick, MA, USA). The Keysight devices were operated and read out via BenchVue (Keysight Technologies, Santa Rosa, CA, USA). The evaluation was carried out with Microsoft Excel (Microsoft Corporation, Redmond, DA, USA).

## 3. Results 

### 3.1. Biomimetic Joint Assemblies of the Owl’s Neck Spine

**Figure 9:**
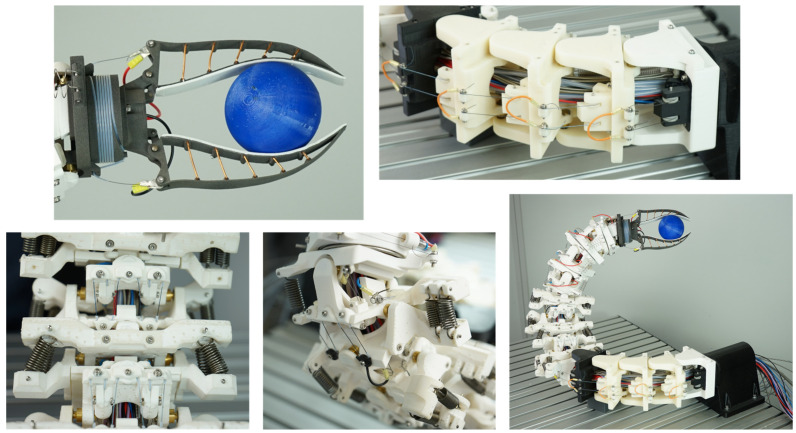
Prototype of the biomimetic owl neck spine and fundamentally different joint assemblies.

In the developed prototype (Figure 9), the SMA wire actuators described in Section 2.3 with diameters of 0.25 mm and 0.381 mm were built into the biomimetic vertebrae of the articulated robot arm described in Section 2.4 and manufactured using the FDM AM technology of Section 2.2. 

With the prototype shown, the requirements of the VDI guideline 6220 [20], which are needed for a biomimetic system, were fulfilled as listed below. The description of the successfully developed prototype using this guideline is presented for general illustration and possible comparison with other biomimetic systems. 

#### 3.1.1. A Biological Model Was Available

As described in Section 2.1, the model of the highly flexible owl neck spine of American barn owls was available. The biologically possible movements and joint arrangements were described in detail by Krings et al. [19,22] and were used as the foundation for the articulated robot arm shown in Figure 9. Approximately, the biological model had a possible head rotation of 270° around the vertical axis and 180° around the horizontal axis [22].

#### 3.1.2. The Working Principle Was Abstracted and Transferred to a Technical Application

Abstracted technical cervical vertebrae were available for the design of an articulated robotic arm modelled on the owl’s cervical spine. The 14 biological cervical vertebrae, which were lined up and primarily linked with saddle joints, consisted of 23 rotary joints in the technical version. In all cases, the rotary joints consisted of brass shafts and ball bearings. This design was chosen due to the lack of technical saddle joints. As muscle substitutes, SMA wire actuators were installed as agonist and different tension springs as antagonist. Simulation results in the form of reachability maps [18] were available to simulate the theoretical range of motion. In addition, the supply channels found in biology in the centre of the vertebrae were technically replicated and used to install the electrical supply lines and the SMA wire actuators.

#### 3.1.3. A Prototype of the Implementation Was Available

**Figure 10:**
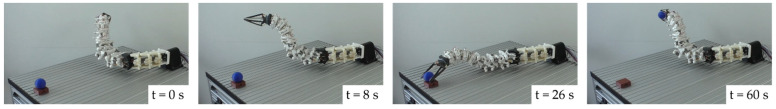
Movement sequence for pivoting, tilting, and gripping a sphere. The duration of the entire sequence was 60 s.

Due to the described abstracted working principle of the individual joint assemblies and the simulation of the range of motion to control the prototype, the natural motions of the owl’s neck spine could be executed. Movements could be performed over almost the entire working area of 900 mm × 800 mm and objects with different geometries could be grasped. The articulated robot arm was controlled by the simulation of inverse kinematics [23], which always utilises the minimum or maximum limit stop positions of the individual technical vertebrae. The number of 23 technical vertebrae, each with two possible limit stop positions, ensured sufficient freedom of movement, as described in [18]. A complex control of the SMA wires to hold intermediate positions of the individual technical vertebrae was, therefore, not necessary. The target point of the gripper to be reached was determined and the inverse kinematics calculated the individual positions of the individual technical vertebrae. This inverse kinematics was based on the simulation described in [18]. A control system to track the actual pose of the robot arm has not yet been implemented in the described prototype. Figure 10 shows, in four pictures, an exemplary movement sequence for picking up a spherical object. In this movement, the use of the three significantly different joint assemblies, namely the roll movement in the lower area, the pitch movement in the middle area, and the gripping movement, can be seen very clearly. These three biomimetic joint assemblies served as models for the technically abstracted joint assemblies in the test rig. In addition to this, another main movement was performed for the rotation of the Fin Ray gripper by the yaw movement in the upper area of the articulated robot arm.

### 3.2. Technical Joint Assemblies and Experimental Results

In the following, the experimental results of the technical joint assemblies from Section 2.5 are described and evaluated. The experiments were carried out with the components and parameters listed in Table 3. An important influence on the coupled force and displacement measurements was the tension spring balance used. Due to the linear spring constant of 0.25 N/mm, a higher force always occurred with increasing shortening of the SMA wire actuator. An experimental setup with a longer integrated wire and thus a greater absolute shortening resulted in a higher measured force.

#### 3.2.1. Straight Version

**Figure 11:**
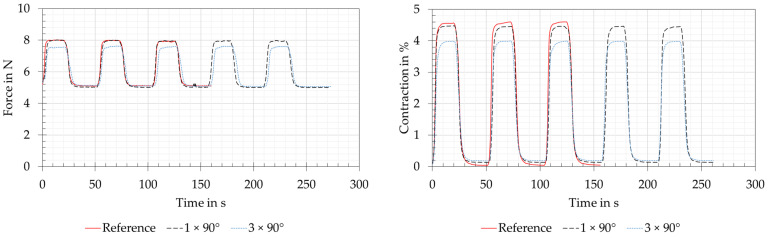
Straight and deflection versions: force and contraction measurements for the reference measurement on the straight clamped SMA wire actuator, with the deflection by 1 × 90° and 3 × 90°. The wire length was 250 mm for all deflection versions, and measurements are taken with 5 N.

The force and contraction measurements of the straight version are shown as red lines in Figure 11. This version was the reference measurement with a straight piece of clamped wire, without deflection. The set preloading force of 5 N and the force of 7.9 N, which could be read off the tension spring balance at the specified power, are shown with slight deviations on the left-hand side of Figure 11. The measurement of the wire contraction is shown on the right side of Figure 11 and was approximately 4.6% for a full phase transformation of the SMA. The assumption for the full phase transformation refers to the plateaus seen when maximum force and contraction are reached. The measurements also appeared plausible due to the mathematically correct measured force difference of 2.875 N at a shortening of 4.6% (11.5 mm) with a spring constant of 0.25 N/mm of the tension spring balance. After each run, the SMA wire actuator also returned to its initial shape at 0% shortening and 5 N preloading force.

#### 3.2.2. Deflection Version

The force and displacement measurements of the joint variants of the deflection version are shown in Figure 11 for direct reference with the straight version. This is due to the fact that the wire lengths and electrical power ratings were the same (see also Table 3). The force measurements yielded almost identical results to the reference for the variant with “1 × 90°” deflection and the measured force decreases by 0.4 N for the variant “3 × 90°”. The contraction measurements of the “1 × 90°” and “3 × 90°” variants showed losses of 0.1% and 0.6%. At the same time, a small remaining contraction of 0.1–0.2% remained with both variants. As with the straight version, plateaus appeared in the contraction measurement in the powered state, from which a completed phase transformation of the wire actuators could be concluded. The force and contraction losses resulted from friction and temperature losses in the deflection points and therefore increased with an increasing number of deflection pulleys. 

#### 3.2.3. Helix Version

**Figure 12:**
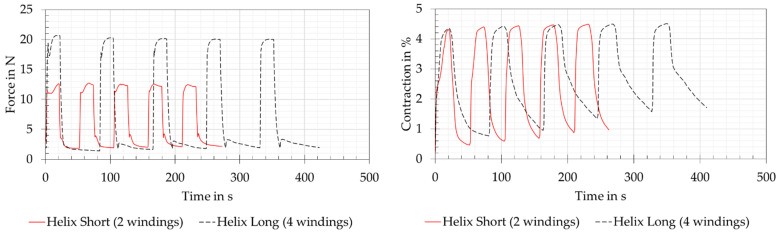
Helix version: force and contraction measurements for the short and long helix variants. The wire length was 450 mm for the short helix and 750 mm for the long helix. The short helix was supplied with 7 V and 0.9 A and the long helix with 14 V and 1.1 A. Both versions were preloaded with 5 N.

The force and contraction measurements of the joint variants of helix version are shown in Figure 12. It should be noticed that the cooling time of the long helix version doubled from 30 to 60 s and that the electrical performance data increased by factors of 1.66 (short helix) and 2.44 (long helix). In the force measurements of the short helix and long helix variants, no direct comparison could be made with the reference measurement of the straight version due to the mechanically very different design and the different wire lengths of 450 mm and 750 mm. However, it was possible to compare the values measured with the load cell with the analogue force display values of the tension spring balance. When supplied with power, the force displayed on the tension spring balance was 9.7 N for the short helix variant and 12.5 N for the long helix variant. In both variants, a strongly increased force was measured at the load cell, which was due to the strong friction losses within the SMA wires with PTFE tubing wrapped helically around a base body. When the measured force increased, both variants initially had a peak that dropped again and then rose to the final force value. This can be explained by the inhomogeneous and thus time-delayed shortening of the air-exposed wire sections or those covered by the PTFE tube and by the friction within the tube. It can be assumed that the exposed section of the SMA wire attached to the load cell first contracted and, at the same time, due to the friction within the tube, could only pull the tension spring balance with a high degree of friction. The open wire section underneath the tube contracted at the same time, but had little influence on the load cell’s force measurement. After a short time, the SMA wire in the tube was also heated up and the full contraction could be measured, but with increased force measurement due to the friction within the tube system. This assumption was confirmed for both variants, as the tube systems were heated up with increasing measurement cycle repetitions and the effect, therefore, no longer occurred in the fifth measurement cycle. The measured contractions of the two variants showed almost 4.5%, which corresponds to the reference measurement. However, this contraction could only be achieved by the increased electrical power mentioned at the beginning of this section, as the tube system in combination with the base body acted as a large heat sink for the SMA wire. The general heating of the system resulted in a loss of dynamic performance of the actuator system. At the same time, an increase in the maximum contraction and a not complete relaxation of the wires could be observed with an increasing number of cycles. This could be explained by the heating of the tube system.

#### 3.2.4. Pulley Block Version

**Figure 13:**
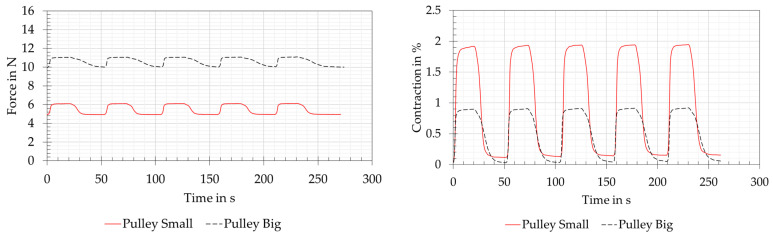
Pulley block version: force and contraction measurements for the small and big pulley variants. The wire length was 250 mm for the small pulley and 450 mm for the big pulley. The small pulley was supplied with 3 V and 0.7 A and was preloaded with 5 N. The big pulley was supplied with 5.4 V and 0.7 A and preloaded with 10 N.

The force and contraction measurements of the joint variants of pulley block version are shown in Figure 13. These two variants were supplied with electrical power in the matching factor, related to the wire lengths, for the reference measurement. It should be noticed that a doubled preload force of 10 N was used for the big pulley variant, as otherwise a total reset of the system to the preload force of 5 N was not achievable. For the small pulley variant, the preload force remained at 5 N, analogous to the reference measurement. The forces that could be read off the tension spring balance when powered are 6.1 N for the small pulley and 11 N for the big pulley. Both force measurements were carried out with the load cell showing very similar results and therefore seemed plausible. The small differences in force between the preload force and the maximum force resulted from the pulley arrangement, whereby the contraction of the system decreased as a factor of the number of moving wires. This could be clearly seen in the contraction measurements. In combination with friction losses due to the multiple consecutive 180° deflections of the SMA wire, analogous to the deflection version, the pulley arrangement resulted in the relatively represented contraction of the system in a factor of two for the small pulley and four for the big pulley. Due to the mostly air-exposed SMA wires, the heating and cooling times were little influenced by the deflection pulleys.

## 4. Discussion

### 4.1. Biomimetic Owl Neck Spine

Compared to existing hyper-redundant and continuum robots, the muscle-like drives in the form of SMA wire actuators stood out. Other systems used, for example, were cable-driven systems with conventional electrical motors [31,32]. In this case, the cables had to be routed through the inside of the robot arm, analogous to the Bowden cables of the biomimetic owl’s neck spine. This resulted in a massive base body to which the corresponding motors for the cable were attached. Another drive option was the direct use of electric motors, which were installed directly in the robot arm. This can be demonstrated using the example of snake robots, which are also classified as hyper-redundant robots due to their large number of joints [33]. The weight of the directly mounted motors had to be moved as well. Another comparable robot arm is the continuum robot by Mahl et al. [34], which was pneumatically driven and was based on the biological model of an elephant trunk. This system is characterised by its inherent compliance. However, an external pneumatic unit was needed to generate movement. Compared to the aforementioned robotic arms, the biomimetic owl neck spine had an extremely light dead weight of the drives. However, the weight was increased by the Bowden cables used and by the deflections under the work table. Overall, the low material input of the NiTi alloys of the SMA wire actuators used less critical materials than, for example, rare earths in electric motors. This and the lightweight AM components fulfilled the requirement of a resource-efficient system. The disadvantage of this actuator technology is the slow movements of the robot arm. This was caused by the necessary cooling times of the SMA wires, which were further increased by the Bowden cables. The movement in Figure 10, for example, took 60 s. The opening movement for the 50° angle of the gripper took 8 s and the closing to the initial position 30 s in the configuration shown. The time for closing the gripper shortened accordingly with the size of the gripping object because of the shorter displacement of the gripper jaws.

### 4.2. Technical Joint Assemblies

When the SMA wire with a diameter of 0.25 mm was clamped straight (straight version) as a reference measurement, the contraction of 4.5% specified by the manufacturer [14] was not only achieved as expected, but even slightly surpassed. After each cycle, the SMA wire returned to its initial shape with 0% contraction. These tests confirmed the suitability as a reference measurement. 

The use of deflection pulleys (deflection version) reduced the contraction of the system. As the number of pulleys increased from one to three, the contraction further decreased. This was also observed in fatigue tests by Mohd Jani [15]. It can be expected that the force and contraction values of the reference measurement can be achieved with a higher electrical power and thus a higher energy consumption.

The contraction of 4.5% achieved with the helix version, despite noticeable friction losses, as described in Section 3.2.3, being contrary to the experimental results of [35]. There, a loss of 35% of the contraction was provided when the wire was wound 360°. This probably occurred because of the higher force of 100 N for a wire diameter of 0.7 mm. The entire system with the SMA wire in the PTFE tube, which was wrapped around the AM manufactured body, acted as a heat sink. Therefore, more electrical energy had to be applied to heat up the SMA wire. This observation and a general heating of the system was confirmed by the measurements of [35]. However, this design made it possible to fit a larger contraction into a more compact installation space. This is confirmed by the test results of Helps et al. [36], which show an increased shortening of 69.81% in the same installation space compared to a straight wire.

In the pulley block version, reduced contractions could be measured by a factor of two for the small pulley and by a factor of four for the big pulley. The load capacity of SMA wires in such an arrangement increased analogously by the same factors. The use of such pulleys to increase the force in the SMA system was recommended by [15] and applied by [37] to develop a vacuum gripper. This vacuum gripper worked with a tension spring to generate the vacuum and with the SMA wires arranged in a circle resembling a pulley system to generate the necessary reset force. Based on the own test results and the described successful applications of other researchers, this pulley variant can be used for joint variants with high-force requirements at a low contraction.

In summary, the designer of such technical joint assemblies must select the most suitable, described variant, consider its advantages and disadvantages, and integrate it into the final system.

## 5. Conclusions and Outlook

This article presented the development of additively manufactured joint assemblies with SMA wire actuators as drive technology. Additive manufacturing is mainly characterised by the possibility of a geometrically flexible design. The SMA wire actuators offer the highest energy density among the known actuator technologies [16,17]. With the combination of these potentials, it was possible to integrate large wire lengths into compact joint assemblies and thus develop actuator assemblies with large strokes, acceptable forces, and high resource efficiency. The basic design ideas for the joints originated from a biomimetic project to develop an articulated robot arm modelled on the American barn owl. This involved working on the problem of fitting large SMA wire lengths into compact biomimetic owl neck vertebrae.

Using an additively manufactured prototype of the biomimetic owl neck spine with integrated SMA wire actuators as muscle substitutes, the basic function of the flexible movement could be reproduced. The 14 biological cervical vertebrae, most of which occur as saddle joints, were abstracted as 28 technical rotary joints and finally realised as 23 rotary joints. 

With the demonstrated function of the biomimetic owl neck as a basis, further abstracted technical joints were developed and their basic function and basic performance data (force and contraction) were first examined with the help of a test rig within the framework of preliminary tests. The basic function of all technical joints was thus proven. This made it possible to integrate longer wire lengths of SMA actuators into more compact design volumes. It should be noted that a larger number of deflection points or windings resulted in increased force and contraction losses. In addition, it is known that material fatigue and thus a shortened lifetime can be expected [15,38].

The test rig used will be upgraded in the form of a frictionless bearing and a non-contact displacement measurement system in order to be able to continue the development of the joint assemblies. This is intended to further quantify the measurements described in this article in order to optimise friction and temperature losses in the deflection points. In addition, the joint assemblies will be further developed with regard to a lightweight design and the resulting resource efficiency, e.g., through topology-optimised AM carrier components, and the use of further AM manufacturing technologies, e.g., SLM. In addition, the findings of the measurements carried out on the joint assemblies will be made available to other researchers and engineers within the framework of a design guideline with design catalogues. This should enable shorter and thus more efficient development processes for innovative SMA + AM joint assemblies.

## Figures and Tables

**Table 1:** Manufacturing parameters and relevant material characteristics of ABS-M30i and SR30 Support of the FDM printer Stratasys Fortus 400mc Large [24,25].

Manufacturing Parameters	ABS-M30i	SR30 Support
Tip (Nozzle)	T10	T12SR30
Slice Height (mm)	0.127	
Part Interior Style	Solid	
Support Style		Sparse
**Material Characteristics**		
Tensile Strength XZ-Orientation (N/mm^2^) ^1^	31	
Tensile Strength ZX-Orientation (N/mm^2^) ^1^	26	
Heat Deflection Temperature @ 0.45 N/mm^2^ (°C) ^2^	96	
Heat Deflection Temperature @ 1.80 N/mm^2^ (°C) ^2^	82	
Glass Transition Temperature (°C) ^3^	108	

Test Methods: ^1^ ASTM D638, ^2^ ASTM D648, ^3^ DSC (SSYS).

**Table 2:** Application-related characteristics of the SMA wire actuators used [14,26].

Characteristics	Flexinol LT 0.254 mm (0.01 in)	Flexinol LT 0.381 mm (0.015 in)
Resistance (Ω/m)	18.5	8.3
Maximum Pull Force (N)	8.7	19.7
Approximate Contraction (%)	4.5	
Approximate Current at Room Temperature (mA)	1050	2250
Recommended Power (W/m)	20.0	60.5
Austenite Start Temperature A_s_ (°C)	68	
Austenite Finish Temperature A_f_ (°C)	78	
Martensite Start Temperature M_s_ (°C)	52	
Martensite Finish Temperature M_f_ (°C)	42	
Contraction Time (s)	1	
Approximate cooling Time at 70 °C (s)	5.4	10.5

**Table 3:** Description of the deflection versions and variants installed in the test rig. If there are several values per line, the values of the following line always refer to the order of the previous ones. If only one value is listed below, it applies to both previous values.

Characteristics	Straight Version	Deflection Version	Helix Version	Pulley Block Version
Wire length (mm)	250	250	750 450	450 250
Wire guide (Variants)	/	3 × 90° 1 × 90°	4 × windings 2 × windings	4 × movable wires 2 × movable wires
Guide material	/	Pulley (PTFE)	Tube (PTFE)	Pulley (PTFE)
Electrical current (A)	0.7	0.7	1.1 0.9	0.7 0.7
Electrical voltage (V)	3	3	14 7	5.43
Mechanical preload (N)	5	5	5	10 5
Time under power (s)	20	20	20	20
Cooling time (s)	30	30	60 30	30

## Data Availability

Further information and videos on the biomimetic project of the technical owl neck spine can be found following the following links: https://baybionik.de/teilprojekte/p8-eulenhalsgelenk/ (accessed on 12 December 2022) https://www.youtube.com/watch?v=3Cl38fQV76g (accessed on 12 December 2022).
